# A *Helicobacter pylori* Homolog of Eukaryotic Flotillin Is Involved in Cholesterol Accumulation, Epithelial Cell Responses and Host Colonization

**DOI:** 10.3389/fcimb.2017.00219

**Published:** 2017-06-06

**Authors:** Melanie L. Hutton, Kimberley D'Costa, Amanda E. Rossiter, Lin Wang, Lorinda Turner, David L. Steer, Seth L. Masters, Ben A. Croker, Maria Kaparakis-Liaskos, Richard L. Ferrero

**Affiliations:** ^1^Centre for Innate Immunity and Infectious Diseases, Hudson Institute of Medical ResearchMelbourne, VIC, Australia; ^2^Monash Biomedical Proteomics Facility, Monash UniversityMelbourne, VIC, Australia; ^3^Inflammation Division, The Walter and Eliza Hall InstituteMelbourne, VIC, Australia; ^4^Infection and Immunity Program, Monash Biomedicine Discovery Institute and Department of Microbiology, Monash UniversityMelbourne, VIC, Australia

**Keywords:** *Helicobacter pylori*, lipid rafts, membrane raft domains, cholesterol, bacterial flotillins, type IV secretion system, CagA

## Abstract

The human pathogen *Helicobacter pylori* acquires cholesterol from membrane raft domains in eukaryotic cells, commonly known as “lipid rafts.” Incorporation of this cholesterol into the *H. pylori* cell membrane allows the bacterium to avoid clearance by the host immune system and to resist the effects of antibiotics and antimicrobial peptides. The presence of cholesterol in *H. pylori* bacteria suggested that this pathogen may have cholesterol-enriched domains within its membrane. Consistent with this suggestion, we identified a hypothetical *H. pylori* protein (HP0248) with homology to the flotillin proteins normally found in the cholesterol-enriched domains of eukaryotic cells. As shown for eukaryotic flotillin proteins, HP0248 was detected in detergent-resistant membrane fractions of *H. pylori*. Importantly, *H. pylori HP0248* mutants contained lower levels of cholesterol than wild-type bacteria (*P* < 0.01). *HP0248* mutant bacteria also exhibited defects in type IV secretion functions, as indicated by reduced IL-8 responses and CagA translocation in epithelial cells (*P* < 0.05), and were less able to establish a chronic infection in mice than wild-type bacteria (*P* < 0.05). Thus, we have identified an *H. pylori* flotillin protein and shown its importance for bacterial virulence. Taken together, the data demonstrate important roles for *H. pylori* flotillin in host-pathogen interactions. We propose that *H. pylori* flotillin may be required for the organization of virulence proteins into membrane raft-like structures in this pathogen.

## Introduction

The human gastric pathogen *Helicobacter pylori* induces chronic gastric inflammation that usually remains asymptomatic. In 10–20% of infections, however, individuals develop either peptic ulceration or gastric cancer (The EUROGAST Study Group, 1993). These severe forms of disease are more commonly associated with infection by *H. pylori* strains which harbor a *cag* pathogenicity island (*cag*PAI), encoding a type IV secretion system (T4SS) (Montecucco and Rappuoli, 2001). The *H. pylori* T4SS system mediates the induction of pro-inflammatory (e.g., interleukin-8, IL-8) responses (Viala et al., 2004) and a cell scattering or so-called “hummingbird” phenotype in epithelial cells (Segal et al., 1999). These responses are mediated by the T4SS-dependent delivery of cell wall peptidoglycan (Viala et al., 2004) and the effector protein, CagA (Odenbreit et al., 2000), respectively. In contrast, the *H. pylori* T4SS appears to be dispensable for the induction of cytokine responses in macrophages and monocytes (Maeda et al., 2001; Gobert et al., 2004; Koch et al., 2016).

*H. pylori* T4SS functionality depends on cholesterol-rich microdomains in the plasma membrane of epithelial cells (Lai et al., 2008; Hutton et al., 2010). These microdomains are known as membrane rafts, also commonly referred to as lipid rafts. Interestingly, cholesterol is an important factor for *H. pylori* chemotaxis and adherence (Ansorg et al., 1992). *H. pylori* has a specific affinity for cholesterol (Trampenau and Muller, 2003) and is able to grow in cholesterol-supplemented media (Testerman et al., 2001). This is consistent with the fact that *H. pylori* does not appear to carry cholesterol biosynthesis genes critical for *de novo* sterol synthesis (Testerman et al., 2001) and must obtain the cholesterol from an exogenous source. Indeed, *H. pylori* is able to up-regulate cholesterol gene expression in gastric epithelial cells *in vitro* (Guillemin et al., 2002), suggesting one mechanism by which the bacterium may ensure an abundance of cholesterol is present in its environment. *H. pylori* can acquire cholesterol from membrane raft domains in host cells for incorporation into its own membrane (Wunder et al., 2006). Once incorporated, cholesterol is α-glucosylated by a cholesterol α-glucosyltransferase (Wunder et al., 2006), resulting in glycolipid forms called cholesteryl glucosides. This α-glucosylation of cholesterol allows *H. pylori* to escape phagocytosis, T-cell activation and bacterial clearance *in vivo* (Wunder et al., 2006), thereby providing a novel mechanism for persistence within the host.

Cholesterol is an indispensable constituent of the plasma membrane and is required for many functions in eukaryotic cells, including cell viability, proliferation (Goluszko and Nowicki, 2005), and for the formation of membrane rafts (Simons and Ikonen, 1997). Membrane rafts control numerous protein-protein and lipid-protein interactions at the cell surface and have been implicated in protein sorting, membrane trafficking, cholesterol metabolism, and signal transduction events (Simons and Toomre, 2000; Manes et al., 2003). Prokaryotes may also contain membrane domains with the characteristic structural and functional features of membrane rafts (Lopez and Kolter, 2010). The membrane raft domains in bacteria are likely to harbor and organize proteins involved in small molecule translocation, protein secretion and signal transduction. These membrane raft-like domains have been identified in the human pathogen *Borrelia burgdorferi* and are thought to contribute to the pathogenesis of Lyme disease (Larocca et al., 2010; Toledo et al., 2014).

Eukaryotic membrane rafts typically contain many proteins, including a prominent raft-associated protein called flotillin, also known as reggie (Simons and Toomre, 2000). There are two known flotillin proteins: flotillin-1 (reggie-2) and flotillin-2 (reggie-1), both of which associate with membrane rafts (Lang et al., 1998). Flotillin-1 is involved in a variety of cellular processes, including vesicle trafficking, cytoskeletal rearrangement, and signal transduction (Langhorst et al., 2005). Flotillin proteins also play key roles in cell-cell adhesion (Otto and Nichols, 2011), clathrin-independent endocytosis (Otto and Nichols, 2011), and the uptake of dietary cholesterol via vesicular endocytosis (Ge et al., 2011).

Flotillins belong to the Stomatin, Prohibitin, Flotillin, and HflK/C (SPFH) protein superfamily, whose members share an SPFH domain at their N-terminus. These proteins are highly conserved across human and animal species and also exist in some bacteria, plants and fungi (Langhorst et al., 2005). Bioinformatic analyses indicate that most bacterial genomes encode proteins with similarity to Flotillin-1 (Lopez and Kolter, 2010). The best characterized of these bacterial flotillins is the YuaG protein of the gut commensal, *Bacillus subtilis*. This flotillin homolog was reported to play roles in a diverse range of cellular functions, including cell division, the maintenance of bacterial shape, and sporulation (Donovan and Bramkamp, 2009; Bach and Bramkamp, 2013; Mielich-Suss et al., 2013). It has been suggested that bacterial flotillins, such as YuaG, may be involved in membrane order or organization and are likely to guide the recruitment of specific proteins to defined areas within the membrane (Bach and Bramkamp, 2013). Consistent with this suggestion, *B. subtilis* YuaG forms punctate staining patterns along the cell membranes of the bacterium (Donovan and Bramkamp, 2009; Lopez and Kolter, 2010) and interacts with proteins involved in various functions, including protein secretion, cell wall metabolism and signaling processes (Bach and Bramkamp, 2013). Although a flotillin homolog has been cloned from the human pathogen, *Staphylococcus aureus* (Lopez and Kolter, 2010), the role(s) of flotillin proteins in bacterial pathogenesis have yet to be investigated.

Herein, we present the first characterization of a flotillin-like protein in the virulence of a human pathogen, *H. pylori*. This protein (HP0248) was shown to be involved in the accumulation of cholesterol within *H. pylori* cell membranes. Importantly, we show that *H. pylori HP0248* mutants were affected in their abilities to induce T4SS-dependent responses in gastric epithelial cells and to establish chronic infection in mice. Collectively, the data demonstrate that the *H. pylori* flotillin-like protein, HP0248, is involved in the accumulation of bacterial membrane cholesterol, thereby contributing to *H. pylori* pathogenesis.

## Materials and methods

### Bacterial strains, media, and culture conditions

*H. pylori* 251 and 26695 are *cag*PAI^+^/T4SS^+^ laboratory strains (Viala et al., 2004), whereas SS1 (*cag*PAI^+^/T4SS^−^) and X47-2AL (*cag*PAI^−^/T4SS^−^) are mouse colonizing strains (Grubman et al., 2010). The *H. pylori* 251Δ*cagA* mutant was described previously (Hutton et al., 2010). *H. pylori* strains were routinely cultured on either blood agar or brain heart infusion broth (BHI; Oxoid) containing a modified Skirrow's selective supplement (comprising vancomycin, 10 μg/ml; polymyxin B, 25 ng/ml; trimethoprim, 5 ug/ml; and amphotericin B, 2.5 μg/ml), according to standard procedures (Ferrero et al., 1998). *Escherichia coli* BL21 was propagated on Luria-Bertani (LB) agar or broth with the appropriate antibiotic. *H. pylori* was incubated with epithelial cells at a multiplicity of infection (MOI) of 10 (Hutton et al., 2010). Viable counts of *H. pylori* were determined by serial dilution and plating. The cholesterol content of the bacteria was determined using the Amplex red cholesterol detection kit, according to the manufacturer's instructions (Molecular Probes, OR, USA).

### Cell culture

Human gastric adenocarcinoma cells (AGS) and murine macrophages (RAW 264.7) were routinely cultured in RPMI medium, supplemented with 10% FCS, 50 units/ml penicillin, 50 μg/ml streptomycin and 1% (v/v) L-glutamine (all reagents from Life Technologies, CA, USA). Cells were seeded at 1 × 10^5^ cells/ml and incubated at 37°C in 5% CO_2_. For co-culture assays, cells were serum-starved overnight in antibiotic-free RPMI medium, then washed 2–3 times in antibiotic-free RPMI medium prior to the addition of *H. pylori* bacteria.

### *In vitro* adherence assay

Bacterial adherence was assessed using modifications to a previously described method (Chionh et al., 2009). Briefly, AGS cells were seeded at 1 × 10^4^ cells in duplicate sets of triplicate wells in 96-well plates (Falcon) and incubated for 24 h at 37°C in 5% CO_2_. Cells were serum-starved overnight and co-cultured with *H. pylori* bacteria for 6 h. One set of the duplicate wells was washed three times with PBS to remove any unattached bacteria. Both sets of wells were fixed for 20 min in a final concentration of 4% paraformaldehyde and then blocked in 1% BSA in PBS for 30 min at room temperature. Cells were incubated for 1 h at room temperature with rabbit anti-*H. pylori* antiserum (diluted 1:500 in PBS; Ferrero et al., 1994). A horseradish peroxidase (HRP)-conjugated goat anti-rabbit antibody (diluted 1:2,000 in PBS; Dako, Glostrup, Denmark) was then added, prior to color development with 3,3′, 5,5′-tetramethylbenzidine (TMB) (Pierce, Thermoscientific, Rockford, IL, USA). The reaction was stopped by adding 0.5 M H_2_SO_4_. Absorbance was read at 450 nm and the percentages of adherent bacteria were calculated by dividing the average OD of the washed set of samples by the average OD of the unwashed set.

### Cytokine responses

Serum-starved cells were stimulated with either live *H. pylori* (MOI = 10) or bacterial lysates (20 μg protein/ml), prepared by freeze-thawing, and incubated at 55°C for 20 min. Following incubation for 1 h, the culture medium was replaced and the cells washed 2–3 times to remove bacteria. Cells were then placed in fresh antibiotic-free medium and incubated a further 5 or 23 h, respectively. IL-8 and IL-6 levels in culture supernatants were determined by sandwich ELISA, according to the manufacturer's instructions (BD Biosciences).

### Isolation of *H. pylori* detergent-resistant membranes (DRMs)

*H. pylori* DRMs were isolated using modifications to a previously described method (Lopez and Kolter, 2010). Briefly, *H. pylori* bacteria (~1–5 × 10^9^ bacteria) were pelleted after 16 h of growth in BHI liquid culture medium, washed three times with 20 mM Tris-HCL (pH 7.5) and resuspended in 20 mM Tris-HCL containing protease inhibitors. Bacterial cells were sheared using a French press and cell debris was eliminated by centrifugation. Membrane fractions were precipitated from supernatants by ultracentrifugation (40,000 × g for 30 min at 4°C) using a Sorvall RC90 ultracentrifuge (Kendro, NC). Proteins that associated with hydrophobic DRM fractions were separated from hydrophilic DSM fractions by phase separation, using the CellLytic MEM protein extraction kit (Sigma) (Lopez and Kolter, 2010).

### Cloning the SPFH domain of HP0248

The Gateway® Cloning System (Life Technologies) was used to generate an expression vector for the production of a His-tag-labeled 163 amino acid region internal to the predicted SPFH domain of *H. pylori* HP0248 (deduced total length 362 amino acids). Primers MH9 and MH10 (Supplementary Table 1) were used to amplify a 487 bp fragment, containing attB1 and attB2 sites, from the *HP0248* gene of *H. pylori* 26695. The resulting PCR product was cloned into the entry clone, pDONR 221. The PCR amplicon was then recombined into the destination vector pDEST17 using LR clonase. Final destination plasmids were confirmed by sequence analysis using the T7 promoter and terminator primers. The pDEST17 expression vector was transformed into *E. coli* BL21 cells for expression.

### Expression and purification of recombinant HP0248

The SPFH domain of *H. pylori* HP0248 was expressed in *E. coli* using standard techniques (see Supplementary Methods for details). Briefly, expression was induced by the addition of 0.4 mM isopropyl β-D-1-thiogalactopyranoside (IPTG; Promega) and the bacterial suspensions pelleted, lyzed by both sonication and 0.1 mg/ml lysozyme (Sigma) treatment. The insoluble pellets were sonicated and the proteins solubilized using 6 M guanidine hydrochloride (Amresco, Ohio, USA). The solubilized proteins were loaded onto a HisTrap HP column (GE Healthcare, Uppsala, Sweden) and recombinant HP0248 purified by immobilized metal ion affinity chromatography. Polyclonal antibodies to HP0248 were generated by administering the purified recombinant protein to a New Zealand White rabbit (Walter and Eliza Hall Institute of Medical Research Antibody Facility; Bundoora, Melbourne, Australia). This anti-HP0248 serum (diluted 1:1,000) was used in Western blot analyses.

### SDS-Page and western blotting

Whole cell lysates, DRM or DSM fractions were resuspended in solubilization buffer, separated in either 12.5% (v/v) acrylamide or 4–12% (v/v) NuPAGE® Bis-tris (LifeTechnologies) gels and transferred to nitrocellulose. *H. pylori* proteins were reacted with rabbit anti-HP0248 serum (diluted 1:1,000) or anti-UreA antibody (1:5,000; Ferrero et al., 1994), followed by addition of a goat anti-rabbit-HRP conjugated antibody (1:1,000 dilution; Dako). Antigen-antibody complexes were detected using ECL detection reagent (Pierce).

### *H. pylori* mutagenesis

Mutagenesis was performed using the Gateway® Cloning System (Life Technologies). Primer combinations and DNA fragments for cloning were as follows: MH1 and MH3 (Supplementary Table 1), a 299 bp fragment from the 5′ end of the *hp0248* of *H. pylori* 26695; GmB4rF and GmB3rR, the gentamicin resistance cassette (Gm^R^) from the pUC1813*apra* vector (Bury-Mone et al., 2003); and MH4 and MH2, a 368 bp fragment from the 3′ end of the *hp0248* gene. PCR products were cloned into three entry clones and integration confirmed by sequence analysis using the M13 primer pair (Supplementary Table 1). Recombination was performed in the destination vector pDEST17 using LR clonase and the final destination plasmid confirmed by sequencing using the T7 promoter and terminator primers (Supplementary Table 1). *H. pylori* Δ*HP0248* mutants were created by natural transformation and selected on horse blood agar (HBA) containing apramycin (30 μg/mL; Sigma) (Grubman et al., 2010). Apramycin-resistant transformants in each strain of *H. pylori* were verified by sequencing using primers MH5, MH6, and GmFwd or GmRvs (Supplementary Table 1). Mutant bacteria were characterized by a 263-amino acid deletion in HP0248. This truncated form of HP0248 included a 185-amino acid deletion within the 231 amino acid SPFH domain.

### Complementation of *H. pylori* HP0248 mutants

Primer combinations and DNA fragments for cloning were as follows: LT8F and LT8R (Supplementary Table 1), a 301 bp fragment from the 5′ end of the *H. pylori* 26695 *rdxA* locus; LT9F and LT9R, a 191 bp fragment, encompassing the *H. pylori* 26695 *ureA* promoter sequence; MH11 and MH12, *H. pylori hp0248* from *H. pylori* 26695; and LT10F and LT10R, the 3′ end of *rdxA*. The four PCR products were cloned into the pDEST17 destination vector and confirmed as above. *H. pylori* Δ*HP0248* mutants, in which an exogenous copy of *hp0248* was inserted into the *rdxA* locus (Smeets et al., 2000), were selected by natural transformation and cultured on HBA plates supplemented with metronidazole (8–32 μg/mL; Sigma). Mutants were verified by sequencing using the primers AG1F, MH5, MH6, and MH13R (Supplementary Table 1).

### Densitometric analysis of HP0248 abundance in DRM and DRM fractions

Western blots of DRM and DSM fractions from *H. pylori* 251 WT, Δ*FLOT*, or *FLOT* (*FLOT*+) strains were analyzed using ImageJ software. The percentage area for HP0248 in each fraction was quantified relative to that for the non-specific protein band in the corresponding fraction. All values were then normalized to the wild-type DRM fraction.

### Proteomic analysis of *H. pylori* DRM and DSM fractions

Coomassie-stained protein bands in DRM samples from *H. pylori* 251 WT were excised from preparative SDS-PAGE gels (4–12% NuPAGE® Bis-tris; LifeTechnologies) and trypsin digested prior to identification by Matrix Assisted Laser Desorption/Ionization Time-of-Flight (MALDI-TOF) and Liquid Chromatography/Mass Spectrometry (LC-MS/MS). For MALDI-TOF, digested samples were co-spotted onto the MALDI target plate and analyzed on an Applied Biosystems (Foster City, CA, USA) 4700 Proteomics Analyser MALDI TOF/TOF in reflectron mode (see Supplementary Methods for details). Data were searched against an in-house database compiled from *H. pylori* genomes downloaded from the ExPASy FTP site (ftp.expasy.org) using the MASCOT search engine (version 1.9, Matrix Science Inc., London, UK) with all taxonomy selected. The following search parameters were used: missed cleavages, 1; peptide mass tolerance, ± 50 ppm; peptide fragment tolerance, ±0.1 Da; peptide charge, 1+; fixed modifications, carbamidomethyl; variable modification, oxidation (Met), and the top five matches reported. Scores were considered significant when above the MASCOT-generated probability-based Mowse score minimum threshold.

LC-MS/MS was performed using an HCT ULTRA ion trap mass spectrometer (Bruker Daltonics, Bremen, Germany), coupled online with an RSLC nano HPLC (Ultimate 3000, Dionex Corporation, SunnyBrook, CA, USA; see Supplementary Methods for details). Data from LC-MS/MS analysis were exported in the Mascot generic file format (^*^.mgf) and searched against an in-house database, as above. The following search parameters were used: enzyme specificity, trypsin; missed cleavages, 1; peptide mass tolerance, ±0.6 Da; peptide fragment tolerance, ±0.3 Da; peptide charge, 2+ and 3+; fixed modifications, carbamidomethyl; variable modification, oxidation (Met).

To identify proteins in DRM and DSM fractions from *H. pylori WT*, Δ*FLOT*, and Δ*FLOT (FLOT*+*)* bacteria, sections of SDS-PAGE gel corresponding to a molecular weight of ~40 kDa were excised. These sections of gel (~1 cm high and spanning the width of the gel) were sliced into six pieces and each trypsin digested prior to analysis. In this case, LC-MS/MS was performed using the QExactive mass spectrometer (Thermo Scientific, Bremen, Germany) coupled online with an RSLC nano HPLC (Ultimate 3000, Thermo Scientific, Bremen, Germany; see Supplementary Methods for details). For each sample, pooled data from six gel slices were exported in the Mascot generic file format (^*^.mgf) and analyzed as above.

### Quantitation of cell scattering responses by high throughput analysis

AGS cells were seeded on coverslips at 1 × 10^5^ cells/ml and incubated for 24 h at 37°C in 5% CO_2_. Cells were then serum-starved overnight prior to co-culture with *H. pylori* for 6 h. Immunofluorescence staining of cellular actin using Alexa Fluor® 488 phalloidin (1:40 dilution; Molecular Probes) was performed as described previously (Hutton et al., 2010). For staining of bacteria, co-cultured cells or *H. pylori* that had been air dried and fixed onto microscope slides (Polysine™, Menzel-Glaser, Braunschweig, Germany) were incubated with rabbit anti-*H. pylori* sera (diluted 1:5,000 in PBS) for 1 h at room temperature, followed by incubation with an anti-rabbit Alexa Fluor® 568 conjugated antibody (1:1,000 dilution; Molecular Probes). The stained bacteria were examined using a Nikon C1 confocal microscope. Actin staining in cells was viewed using the Cellomics ArrayScan VTI HCS Reader (Thermo Scientific), capturing 20 fields per well with the 20 x objective.

### Quantification of CagA translocation by immunofluorescence

AGS cells were seeded in μ-slide eight well-chambers (Ibidi) at 3 × 10^4^ cells/ml and incubated for 24 h at 37°C in 5% CO_2_. Cells were then serum-starved overnight prior to co-culture with *H. pylori* for 6 h. Cells were subsequently fixed using 4% paraformaldehyde and their nuclei stained with DAPI (diluted 1:1,000 in PBS; Molecular Probes). Extracellular CagA was detected using a rabbit anti-CagA (b-300) antibody (1:100; sc-25766, Santa-Cruz Biotechnology), followed by incubation with an anti-rabbit Alexa Fluor 488 conjugated antibody (1:1,000 dilution; Molecular Probes). Cells were then re-fixed and permeabilized using 1% Triton-X. Intracellular CagA was probed using the same rabbit anti-CagA antibody, followed by incubation with an anti-rabbit Alexa Fluor 647 conjugated antibody (1:1,000 dilution; Molecular Probes). Cells were imaged with the same background intensity settings on a Deltavision API wide-field microscope (60 x objective). Image analysis was performed using ImageJ, where relative intensities of A_647_/A_488_ were quantified for five cells per field viewed (five fields viewed per sample; *n* = 2 experiments).

### Mouse infection

Animal handling and experimentation was performed in accordance with Victorian State Government regulations and approved by the Monash University Animal Ethics Committee (ethics no. MMCA 2010/18). *H. pylori* suspensions for mouse inoculation were prepared by harvesting bacteria from HBA plates using BHI broth (Ferrero et al., 1998). Six- to eight-week-old female specific pathogen*/Helicobacter*-free C57BL/6 mice were each intragastrically administered a single 100-μl aliquot of the inoculating suspension (10^7^ cfu/mouse) using polyethylene catheters (Ferrero et al., 1998). The presence of *H. pylori* infection in mice was determined after 30 days of infection by quantitative culture as described previously (Ferrero et al., 1998).

### Statistical analysis

Data were analyzed using the Student's *t*-test, Mann-Whitney *U*-test or ANOVA, as appropriate. Differences in data values were considered significant at a *P* < 0.05.

## Results

### *H. pylori* has a flotillin-like protein (HP0248) that partitions to membrane raft-like domains in its cell membrane

Studies have recently described the presence of membrane raft-like domains within the cell membranes of a pathogenic bacterium, *B. burgdorferi*, suggesting that membrane rafts may be conserved amongst both prokaryotic and eukaryotic organisms (Larocca et al., 2010; Toledo et al., 2014). Consistent with this suggestion, the commensal bacterium *B. subtilis* was reported to contain a protein, YuaG, with homology to flotillins, a family of membrane raft-associated proteins (Donovan and Bramkamp, 2009). A flotillin-like protein was identified in the bacterial species, *S. aureus*, but this protein was not characterized (Lopez and Kolter, 2010). Given that *H. pylori* cell membranes are highly enriched in cholesterol, a feature of membrane rafts, we speculated that *H. pylori* may have a flotillin-like protein. To address this question, we used the primary amino acid sequence of *B. subtilis* YuaG (Donovan and Bramkamp, 2009; Lopez and Kolter, 2010) to perform BLAST analyses on the order *Campylobacterales*. We first identified homologs in *Helicobacter hepaticus* (HH0856) and several *Campylobacter* spp. These proteins were, in turn, used to identify the presence of a hypothetical protein, HP0248, in the *H. pylori* 26695 strain. *hp0248* and its predicted protein in this *H. pylori* strain have accession numbers NC_000915.1 (257084-258172) and NP_207046.1, respectively. We identified a 231 amino acid region in *H. pylori* HP0248, which when subjected to CLUSTALW analysis, exhibited 14.7% identity and 42.7% similarity with the SPFH domain of human flotillin-1 (Supplementary Figure 1) (Langhorst et al., 2005). The SPFH domain of *H. pylori* HP0248 shares a similar level of identity with that of human flotillin-2 (data not shown). Homologs of HP0248 are present in other *H. pylori* strains (e.g., J99, B128, G27), as well as in various human and animal *Helicobacter* spp e.g., *Helicobacter acinonychis, Helicobacter felis, Helicobacter mustelae* (data not shown). This highlights the likely importance of HP0248 in *Helicobacter* biology. On the basis of the findings, we have named the *H. pylori* HP0248 protein, “flotillin-like protein” or “FLOT.”

**Figure 1:**
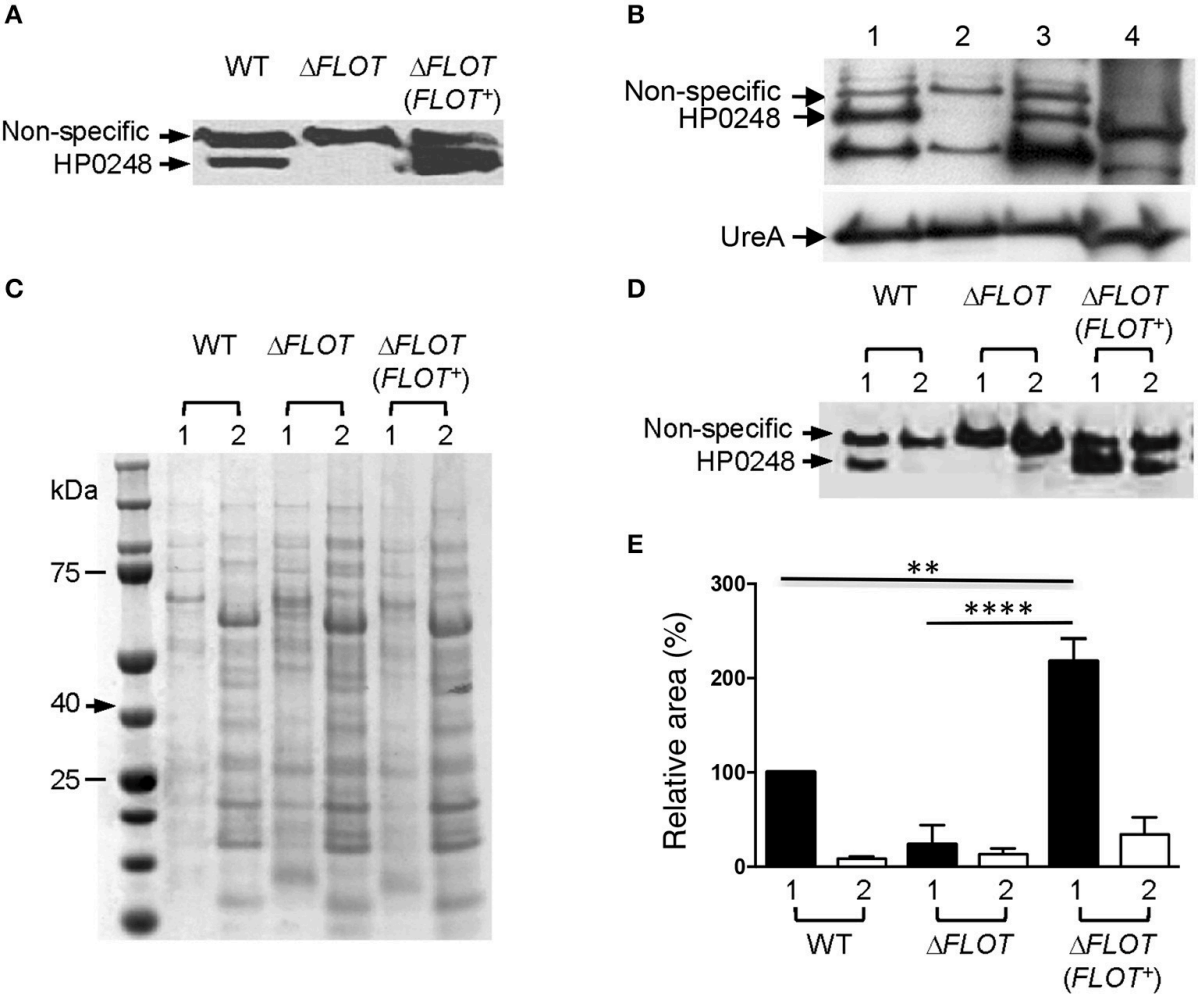
**HP0248 is enriched in the DRM fractions of ***H. pylori*** cell membranes. (A)** Whole cell lysates of *H. pylori* 251 WT, Δ*FLOT*, or *FLOT* (*FLOT*+) bacteria were analyzed by Western blotting. Full length HP0248 protein (molecular weight, *c*. 40 kDa) was detected in WT and *FLOT* (*FLOT*+) preparations, but not in those of the Δ*FLOT* strain. A non-specific protein band was also present in all three preparations. **(B)** Western Blot of *H. pylori* 251 WT whole cell lysate (lane 1), inner membrane (lane 2), outer membrane (lane 3) and cytoplasmic (lane 4) fractions. UreA was used as a loading control. **(C)** Coomassie-stained SDS-PAGE gel showing the total protein profiles of DRM (lane 1) and DSM (lane 2) preparations of *H. pylori* 251 WT. Molecular weight markers are shown. **(D)** Western blot of DRM (lane 1) and DSM (lane 2) preparations from *H. pylori* 251 WT, Δ*FLOT*, or *FLOT* (*FLOT*+) bacteria. **(E)** Densitometric analysis of HP0248 in DRM and DSM fractions from *H. pylori* 251 WT, Δ*FLOT*, and *FLOT* (*FLOT*+) bacteria. The relative amount of HP0248 in each fraction (mean ± SEM from three independent experiments) is expressed relative to that in the WT DRM fraction. Data were analyzed using the one-way ANOVA. ^**^P < 0.01; ^****^P < 0.0001.

In order to characterize the biological functions of the *H. pylori* flotillin-like protein, we generated Δ*hp0248* deletion mutants (Δ*FLOT*) in *H. pylori* 26695 and 251, both of which have functional T4SSs (Viala et al., 2004), as well as in two strains that do not i.e., X47-2AL and SS1 (Grubman et al., 2010). We also generated a complemented mutant (Δ*FLOT* (*FLOT*+)) in the 251 strain. Western blot analyses using a polyclonal rabbit antibody raised against the HP0248 SPFH domain confirmed the production of HP0248 in *H. pylori* 251 wild-type (WT) and Δ*FLOT* (*FLOT*+) strains, but not in a Δ*FLOT* mutant (Figure 1A).

To determine the cellular localization of *H. pylori* HP0248, whole bacteria were fractionated into cytoplasmic, inner and outer membrane compartments (Voss et al., 2014). By Western blotting, HP0248 was shown to be present in the outer but not inner membrane (Figure 1B). The *H. pylori* urease A subunit (UreA), which is present in both cytoplasmic and membrane fractions (Phadnis et al., 1996), was used a sample loading control. Given that flotillins normally partition to membrane rafts, we next sought to determine whether *H. pylori* HP0248 might also localize to membrane raft-like structures within the bacterial membrane. To do this, we exploited the known property of raft-associated proteins to partition to detergent-resistant membrane (DRM) fractions. This approach has been used previously to isolate hydrophobic proteins from membrane rafts in other bacteria (Larocca et al., 2010; Lopez and Kolter, 2010). As shown in Figure 1C, DRM fractions of *H. pylori* membranes exhibited markedly different SDS-PAGE profiles to those of detergent-sensitive membrane (DSM) fractions, consistent with the separation of hydrophobic raft-associated proteins from hydrophilic proteins. Importantly, HP0248 was detected in the DRM fractions of WT and Δ*FLOT* (*FLOT*+), but not in those of Δ*FLOT* bacteria (Figures 1D,E). The presence of HP0248 in the DSM of the Δ*FLOT* (*FLOT*+) mutant can be attributed to over-expression of this protein in the strain due to the use of the strong ureA promoter for complementation (Grubman et al., 2010). Using two different biochemical approaches, we showed that the *H. pylori FLOT* protein associates with the bacterial membrane.

### Proteomic analyses identify *H. pylori* HP0248 within DRM fractions

Proteomic analyses of the total DRM fractions of WT bacteria identified 29 putative and five hypothetical proteins (Table 1). Amongst the putative proteins, 19 were proven experimentally to associate with membranes (Baik et al., 2004; Carlsohn et al., 2006), with an additional four predicted by the PSORTb program to be membrane-associated (Table 1, Figure 1B). Although the six remaining putative proteins are defined as cytoplasmic (Table 1, Supplementary Table 2), five of these have actually been shown to associate with *H. pylori* membranes i.e., HP0072, HP0073, HP0248, HP1462, HP1563. Amongst these predicted cytoplasmic proteins are HP0248 and UreA (HP0073) (Figure 1B, Phadnis et al., 1996). Similar findings were reported in a proteomic study on *B. burgdorferi* DRMs in which the majority (63%) of the proteins found in those fractions were predicted to be associated with the bacterial membrane, however, some cytoplasmic proteins were also detected (Toledo et al., 2015).

**Table 1:** **Proteins identified in DRM fractions of ***H. pylori*** 251 WT bacteria**.

**HP number**	**Protein**	**Predicted protein location^a^**
HP0025	OMP2	Membrane-associated^b^^,^^c^
HP0072	Urease subunit beta (UreB)	Cytoplasmic^b^^,^^c^
HP0073	Urease subunit alpha (UreA)	Cytoplasmic^b^^,^^c^
HP0097	Hypothetical protein	Unknown
HP0127	OMP4 (HorB)	Membrane-associated^b^^,^^c^
HP0130	Hypothetical protein	Unknown
HP0185	Hypothetical protein	Cytoplasmic
HP0229	OMP6 (HopA)	Membrane-associated^b^^,^^c^
HP0248	Flotillin-like protein	Cytoplasmic
HP0252	OMP7 (HopF)	Membrane-associated^b^
HP0317	OMP9 (HopU or BabC)	Membrane-associated^b^
HP0377	Thiol:Disulphide interchange protein (DsbE)	Membrane-associated
HP0472	OMP11	Membrane-associated^b^^,^^c^
HP0486	OMP (HofC)	Periplasmic^b^
HP0606	Membrane fusion protein (MtrC)	Membrane-associated^c^
HP0671	OMP14 (HorF)	Membrane-associated^b^^,^^c^
HP0706	OMP15 (HopE)	Membrane-associated^b^^,^^c^
HP0797	Lipoprotein (HpaA)	Membrane-associated^b^
HP0896	OMP19 (BabB or HopT)	Membrane-associated^b^^,^^c^
HP0912	OMP20 (HopC, AlpA)	Membrane-associated^b^^,^^c^
HP0913	OMP21 (HopB, AlpB)	Membrane-associated^b^^,^^c^
HP1069	ATP-dependent zinc metalloprotease	Membrane-associated
HP1125	OMP18 (Peptidoglycan-associated lipoprotein precursor)	Membrane-associated^b^
HP1132	ATP synthase F1, subunit beta	Cytoplasmic
HP1173	Hypothetical protein	Cytoplasmic^c^
HP1177	OMP27 (HopQ)	Membrane-associated^b^^,^^c^
HP1243	OMP28 (HopS, BabA)	Membrane-associated
HP1395	OMP30 (HorL)	Membrane-associated^b^
HP1462	Secreted protein involved in motility	Cytoplasmic^b^
HP1463	Hypothetical protein	Unknown
HP1469	OMP31 (HopV)	Membrane-associated^b^^,^^c^
HP1488	36 kDa antigen	Membrane-associated
HP1540	Ubiquinol cytochrome c oxidoreductase	Membrane-associated
HP1563	Alkyl hydroperoxide reductase (TsaA)	Cytoplasmic^c^

a *Determined using the PSORTb prediction program (http://www.psort.org/psortb/)*.

b *Membrane association proved experimentally (Carlsohn et al., 2006)*.

c *Membrane association proved experimentally (Baik et al., 2004)*.

To directly compare the protein composition of DRM and DSM fractions, we performed LC/MS-MS analysis on DRM and DSM fractions from *H. pylori* WT, Δ*FLOT* and Δ*FLOT* (*FLOT*+) bacteria. Proteins in the molecular weight range of HP0248 i.e., 40 kDa were selectively analyzed by LC-MS/MS using the QExactive mass spectrometer. This analysis yielded a larger number of protein “hits” than that acquired on the HCT ULTRA ion trap mass spectrometer (Table 1). This is most likely due to the greater level of sensitivity of the former. Most importantly, however, HP0248 was again detected in the DRM but not DSM fractions of WT and Δ*FLOT* (*FLOT*+) bacteria (Supplementary Table 3). Additionally, 50 proteins were identified within the DRM fractions from all three *H. pylori* strains (Supplementary Figure 2), of which 31 were not detected in any of the DSM fractions (Supplementary Tables 3, 4). The latter were considered to be putative membrane raft-associated proteins. In addition to HP0248, several outer membrane proteins (OMPs) were found to partition to the DRM fractions (Table 1, Supplementary Tables 3, 4), suggesting that proteins involved in host-pathogen interactions may selectively localize to the membrane raft-like structures in *H. pylori* membranes. Taken together, the data demonstrate that *H. pylori* HP0248 preferentially associates with a fraction enriched in membrane rafts.

### *H. pylori* HP0248 is important for cholesterol accumulation and induction of IL-8 responses

**Figure 2:**
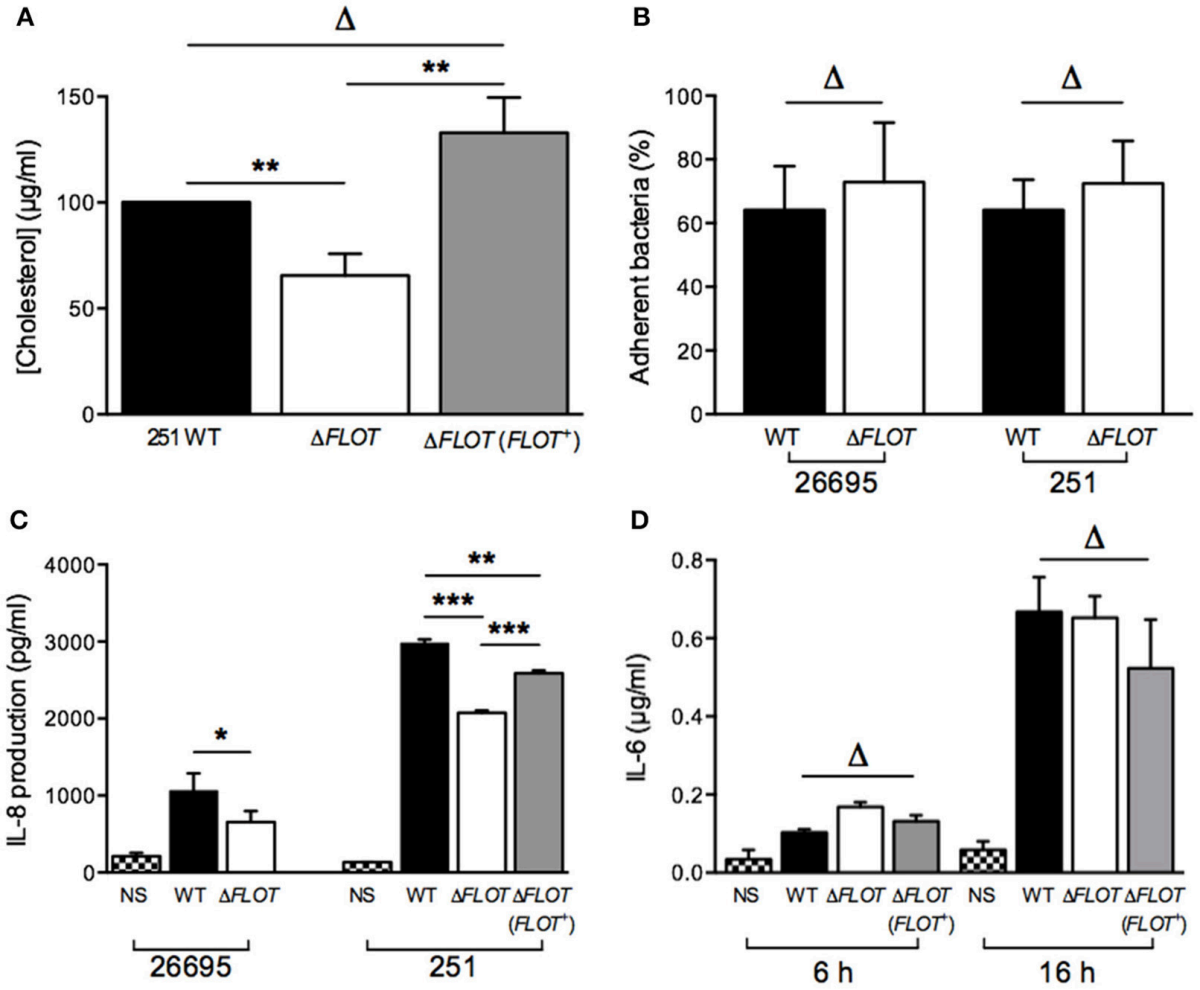
**The ***H. pylori*** flotillin-like protein, HP0248, is important for cholesterol accumulation and induction of cytokine responses in epithelial cells but not macrophages**. **(A)** Levels of cholesterol in *H. pylori* 251 WT, Δ*FLOT*, and Δ*FLOT* (*FLOT*+) bacteria, as measured by the Amplex Red cholesterol detection kit. Data have been normalized to those for the WT strain and thus no error bars can be presented for this group. **(B)** Adhesion assays were performed on AGS gastric epithelial cells that had been co-cultured for 6 h with *H. pylori* WT 26695 or 251 strains, or the corresponding Δ*FLOT* mutants. Data are expressed as the % of bacteria that adhered. The results represent the average of three independent experiments. **(C)** AGS cell responses to *H. pylori* WT, Δ*FLOT*, or *FLOT* (*FLOT*+) bacteria. Non-stimulated cells (NS) served as negative controls. The supernatants were analyzed by ELISA for the detection of IL-8 at 24 h post-stimulation. **(D)** RAW 264.7 cell responses to *H. pylori* 251 WT, Δ*FLOT*, or *FLOT* (*FLOT*+) bacteria. The supernatants were analyzed by ELISA for IL-6, at 6 and 16 h post-stimulation. Data correspond to the mean ± S.E.M. (determined in triplicate) and are pooled from at least three independent experiments. Data were analyzed using the one-way ANOVA **(A,C,D)** or Mann-Whitney test **(B)** ^*^P < 0.05; ^**^P < 0.01; ^***^P < 0.001; Δ*P* > 0.05.

The *H. pylori* 251 Δ*FLOT* and Δ*FLOT* (*FLOT*+) strains grew as well as the WT strain in BHI broth medium supplemented or not with cholesterol (data not shown). Importantly, however, the *H. pylori* 251 Δ*FLOT* mutant strain possessed significantly lower cholesterol levels, when compared with WT bacteria (Figure 2A; *P* < 0.01). Similar findings were obtained for Δ*FLOT* mutants on the *H. pylori* 26695, X47-2AL, and SS1 backgrounds, with these mutants consistently displaying 40% less cholesterol than the respective WT strains (data not shown). Finally, the *H. pylori* 251 Δ*FLOT* (*FLOT*+) complemented strain exhibited WT levels of cholesterol (Figure 2A), thereby confirming the important function of HP0248 in *H. pylori* cholesterol accumulation.

Given the importance of *H. pylori*-associated cholesterol for the induction of cellular responses, we next examined interactions between *H. pylori* WT, Δ*FLOT*, or Δ*FLOT* (*FLOT*+) bacteria and host epithelial cells. As shown in Figure 2B, *H. pylori* Δ*FLOT* 26695 and 251 mutants adhered as well to AGS cells as the corresponding WT strains, suggesting that adhesion occurs independently of the flotillin-like protein. In contrast, *H. pylori* Δ*FLOT* mutant strains on both 26695 and 251 backgrounds induced significantly lower IL-8 responses, when compared with WT strains (Figure 2C; *P* < 0.05 and *P* < 0.001, respectively). Δ*FLOT* (*FLOT*+) bacteria induced significantly increased IL-8 responses when compared with Δ*FLOT* organisms, however, these responses were only partially restored with respect to those induced by WT bacteria (Figure 2C; *P* < 0.001 and *P* < 0.01, respectively). As observed for cholesterol-enriched and cholesterol-depleted bacteria, *H. pylori* Δ*FLOT* mutants induced similar IL-6 and TNF-α responses in macrophages as WT organisms (Figure 2D; data not shown). Cumulatively, these data indicate that *H. pylori* HP0248 is involved in cholesterol accumulation in the bacterial cell membrane and induction of IL-8 responses in epithelial cells. We speculate that the absence of HP0248 may destabilize membrane raft domains in the *H. pylori* cell membrane, thereby affecting T4SS-dependent functions.

### *H. pylori* HP0248 is important for T4SS-induced cell scattering responses and CagA translocation in epithelial cells

**Figure 3:**
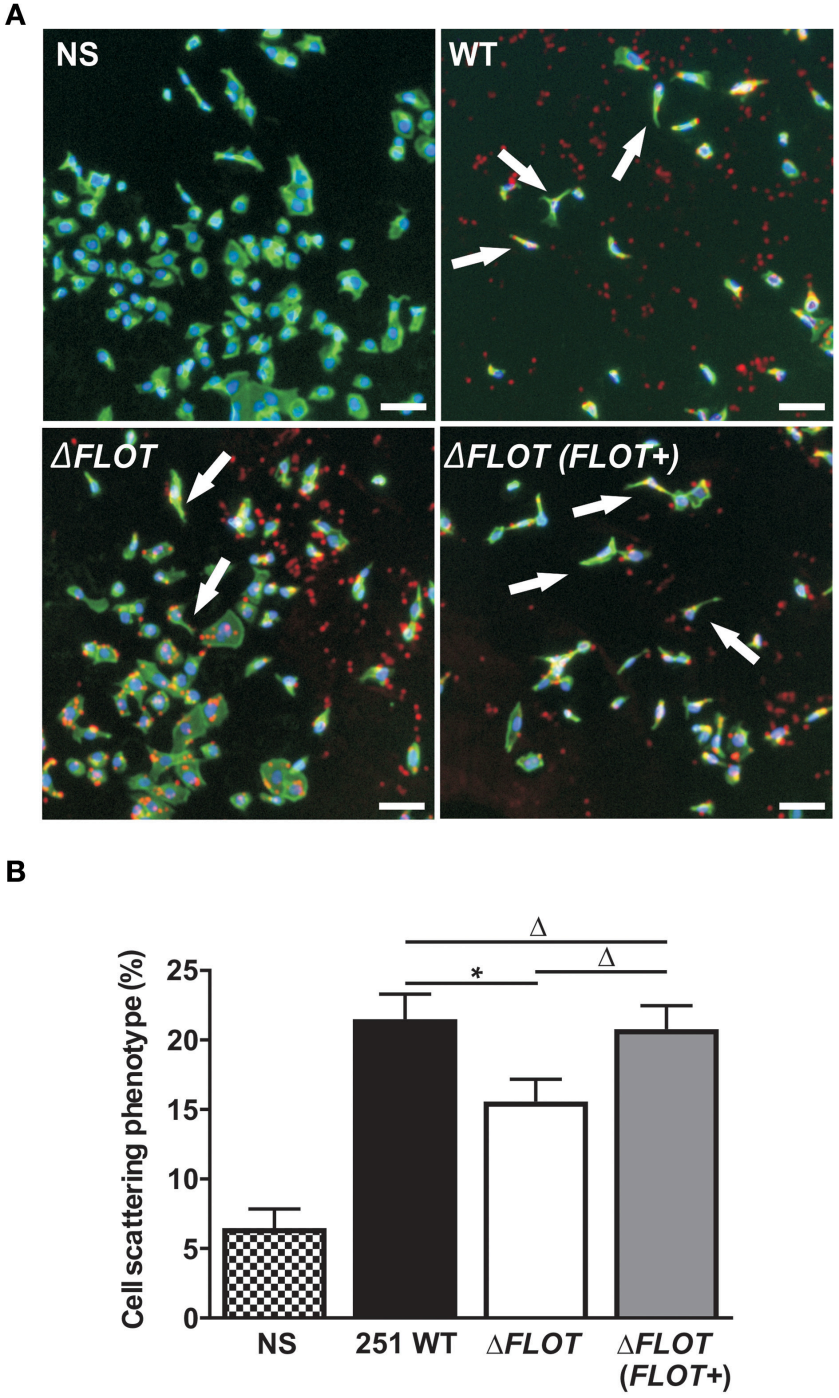
**The ***H. pylori*** flotillin-like protein, HP0248, is important for the induction of cell scattering responses in epithelial cells. (A)** Induction of the cell scattering phenotype in AGS cells, as determined by phalloidin-staining of cells that were either non-stimulated (NS) or co-cultured with *H. pylori* 251 WT, Δ*FLOT*, or Δ*FLOT* (*FLOT*+) bacteria. Cellular actin is stained green, cell nuclei are DAPI stained (blue), and *H. pylori* are stained red. Cellular morphology was examined using the Cellomics Array Scan High Content Screening reader (20 × objective). Arrows indicate cells that are displaying a cell scattering responses or “hummingbird phenotype,” as characterized by cell elongation and the formation of spindles. Scale bars represent 30 μm. **(B)** The percentages of cells displaying cell scattering responses were determined after 6 h co-culture. Cell counting was performed on 20 fields per well for a total of 10 wells per experimental condition, across two independent experiments (with a minimum of 6,500 cells counted per experimental condition). Data correspond to the mean ± S.E.M. from pooled independent experiments (*n* = 2). Data were analyzed using one-way ANOVA ^*^P < 0.05; Δ*P* > 0.05.

In addition to the induction of IL-8 production in epithelial cells, the *H. pylori* T4SS mediates delivery of the effector molecule, CagA, which causes rearrangement of the host cell actin cytoskeleton and results in cell scattering (Segal et al., 1999; Odenbreit et al., 2000; Backert et al., 2001). To further investigate the importance of *H. pylori* HP0248 in T4SS-dependent functions, we co-cultured AGS cells with *H. pylori WT*, Δ*FLOT*, or Δ*FLOT* (*FLOT*+) bacteria, and used a high throughput imaging technique to quantify the proportions of cells displaying a cell scattering response (Figure 3A). Consistent with the IL-8 data (Figure 2C), AGS cells that had been co-cultured with an *H. pylori* Δ*FLOT* mutant strain displayed weaker cell scattering responses, when compared with cells co-cultured with either the WT or complemented mutant strains (Figure 3B; *P* < 0.05 and *P* > 0.05, respectively).

**Figure 4:**
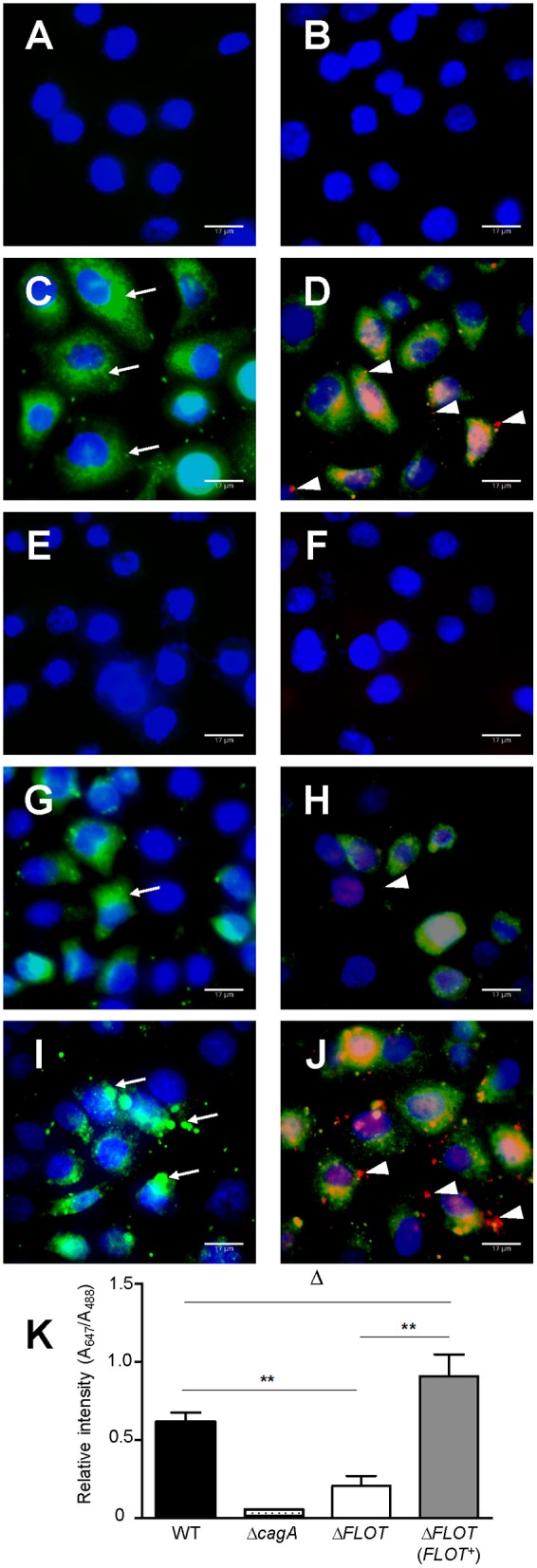
*****H. pylori*** bacteria lacking the flotillin-like protein, HP0248, translocate less CagA into epithelial cells**. AGS cells were either **(A,B)** non-stimulated or co-cultured with **(C,D)** *H. pylori* 251 WT, **(E,F)** Δ*cagA*, **(G,H)** Δ*FLOT*, or **(I,J)** Δ*FLOT* (*FLOT*+) bacteria. CagA was detected using a rabbit anti-CagA antibody. Panels **(A,C,E,G,I)** show cells that had been incubated with anti-rabbit Alexa Fluor 488-conjugated secondary antibody. Panels **(B,D,F,H,J)** show cells that had been incubated with anti-rabbit Alexa Fluor 488-conjugated secondary antibody, permeabilized with 1% Triton-X and then incubated with anti-rabbit Alexa Fluor 647-conjugated antibody. Nuclei (blue) were stained using DAPI. Extracellular CagA molecules (thin arrows) appear as green diffuse areas of staining and were detected using a rabbit anti-CagA (b-300) primary antibody and an Alexa Fluor 488 anti-rabbit conjugate as the secondary antibody. Intracellular CagA molecules (arrow heads) appear as red mainly punctate areas of staining that were detected using the rabbit anti-CagA and Alexa Fluor 647-conjugated anti-rabbit antibodies. Scale bars, 17 μm. *K*, Quantification of Alexa Fluor 647 and Alexa Fluor 488 staining (expressed as relative intensities) in AGS cells that had been co-cultured with either *H. pylori* 251 WT, Δ*cagA*, Δ*FLOT*, or Δ*FLOT* (*FLOT*+) bacteria. The data represent the mean ± SEM for two independent experiments. Data were analyzed using the Mann-Whitney U test. ^**^P < 0.008; Δ*P* > 0.05.

We also confirmed that the reduced levels of cell scattering induced in AGS cells by *H. pylori* Δ*FLOT* bacteria were due to reduced translocation of the CagA effector protein, a functional read-out of T4SS activity. Using an immunofluorescence-based technique to detect both extracellular and intracellular CagA, we observed that the levels of intracellular CagA were significantly reduced in AGS cells that had been co-cultured with *H. pylori* Δ*FLOT* or Δ*cagA* bacteria, when compared with cells co-cultured with either *WT* or Δ*FLOT* (*FLOT*+) bacteria (Figure 4; *P* < 0.02 and *P* < 0.008, respectively). Together, the data show that *H. pylori* Δ*FLOT* bacteria are significantly affected in their ability to induce CagA-dependent cell scattering and IL-8 responses in gastric epithelial cells, thereby confirming the importance of the *H. pylori* flotillin-like protein, HP0248, in T4SS functionality.

### *H. pylori* HP0248 is important for establishment of a chronic infection in mice

**Figure 5:**
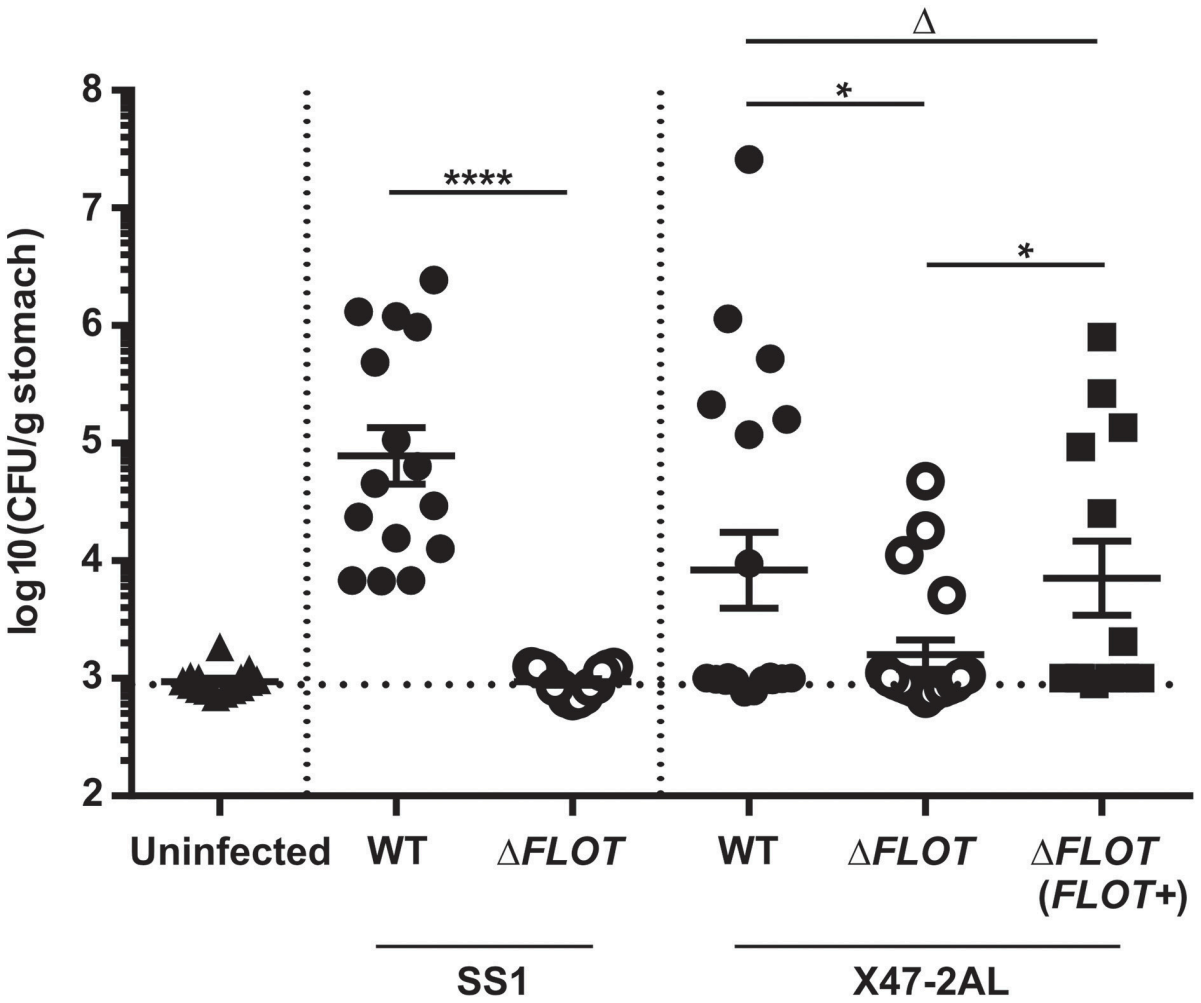
**The ***H. pylori*** flotillin-like protein, HP0248, is required for mouse colonization**. C57BL/6 mice were inoculated with 10^7^ CFU of *H. pylori* by intragastric gavage. Quantitative culture assays were performed at 30 days post-infection. Data are presented as Log_10_colony forming units (CFU)/g stomach, with each point representing the mean of two estimations for a single mouse. Horizontal bars represent the geometric means of viable cell count determinations. The limit of detection for the assay is approximately 1,000 CFU/gram. Data for *H. pylori* SS1 are pooled from three independent experiments, whereas those for X47-2AL are from 2 (WT, Δ*FLOT*) and 1 experiment (Δ*FLOT* (*FLOT*+)), respectively. Data were analyzed using the Mann-Whitney test ^*^P < 0.05; ^****^P < 0.0001; Δ*P* > 0.05.

Finally, mouse infection studies were performed with *H. pylori* Δ*FLOT* mutants that had been generated in the SS1 and X47-2AL mouse-colonizing strains (Grubman et al., 2010). A dramatic and highly significant effect on colonization was observed for *H. pylori* SS1 Δ*FLOT* mutants, with no bacteria cultured from the gastric tissues of these mice at 30 days post-infection (Figure 5; *P* < 0.0001). Although we were unable to complement the *FLOT* mutation in *H. pylori* SS1 (data not shown), we were able to do so in another mouse-colonizing strain, *H. pylori* X47-2AL. Bacterial loads in X47-2AL Δ*FLOT*-infected mice were significantly reduced with respect to those in WT-infected mice, albeit less strikingly than for SS1 (Figure 5; *P* = 0.039). Additionally, mice that were infected with the X47-2AL Δ*FLOT* (FLOT+) strain exhibited significantly higher bacterial loads than animals infected with the Δ*FLOT* mutant (*P* = 0.01), but similar loads to those infected with the WT strain (*P* = 0.549). The fact that the *FLOT* mutation had a more dramatic effect on colonization in SS1 than in X47-2AL is similar to the findings of a previous study (Grubman et al., 2010), indicating likely strain-dependent differences. Taken together, the data demonstrate that HP0248 plays a role in chronic colonization of the mouse gastric mucosa.

## Discussion

It has been known for some time that *H. pylori* obtains host cell-derived cholesterol to generate cholesteryl glucosides which the bacterium incorporates into its own membrane (Ansorg et al., 1992; Hirai et al., 1995). These cholesteryl glucosides allow *H. pylori* to evade phagocytosis, the activation of a T-cell response and thus bacterial clearance (Wunder et al., 2006). We now know that cholesterol plays multiple roles in *H. pylori* pathogenesis. This compound is required during the initial phase of colonization by *H. pylori* (Hildebrandt and Mcgee, 2009), as well as in bacterial resistance to antibiotics, antimicrobial peptides and bile salts (Mcgee et al., 2011; Trainor et al., 2011). In the present study, we have identified an *H. pylori* homolog of eukaryotic flotillin proteins that localizes to the cholesterol-enriched cell membranes of the bacterium. We propose that this *H. pylori* flotillin represents a new virulence factor involved in host-pathogen interactions.

It is well-established that cholesterol accumulates within membrane raft microdomains of eukaryotic cells. An important property of membrane rafts is that they can selectively include or exclude proteins to control interactions at the cell surface (Manes et al., 2003). It is becoming apparent that some bacteria possess domains analogous to eukaryotic membrane rafts in their outer membranes (Larocca et al., 2010; Lopez and Kolter, 2010). Additionally, it has been reported that homologs of the archetypal membrane raft molecule, flotillin, can be found in certain bacteria (Donovan and Bramkamp, 2009; Lopez and Kolter, 2010). For the first time, we have characterized a flotillin-like protein in a human bacterial pathogen and demonstrated its importance in pathogenesis. This protein, encoded by the *hp0248* gene in *H. pylori* 26695, was originally annotated as a hypothetical protein. Consistent with this finding, a recent review article reported *H. pylori hp0248* as being a putative flotillin-encoding gene, however, no evidence for this conclusion was provided (Bramkamp and Lopez, 2015). Interestingly, although we used the amino acid sequence of the *B. subtilis* flotillin-like protein YuaG (also known as FloT) to identify *H. pylori* HP0248, it was suggested that HP0248 was likely to be more similar to the *B. subtilis* flotillin-like protein YqfA (or FloA) (Bramkamp and Lopez, 2015). It is therefore possible that in common with *B. subtilis* and other bacteria, *H. pylori* has two flotillin-like proteins, a fact that may explain the significant but relatively modest differences observed between the *H. pylori* WT and Δ*FLOT* mutants observed here. CLUSTALW analysis of *H. pylori* HP0248 (Supplementary Figure 1) showed that it has a predicted SPFH domain, characteristic of flotillin family members. By homologous recombination, we were able to generate *H. pylori* HP0248 mutants lacking all but 47 amino acids in the N-terminal region of the SPFH domain. In contrast, and despite two different mutagenesis approaches, all attempts to generate *H. pylori* HP0248 mutants lacking the entire SPFH domain were unsuccessful (data not shown). The precise reason for this observation remains unclear. One possibility is that the downstream gene, *hp0249*, which was used as a homology arm for recombination, may be an essential gene for *H. pylori*. The start codon of *hp0249* is situated only seven base pairs from the stop codon of *hp0248* and the two genes are likely to be part of the same transcriptional unit. Thus, any perturbation in *hp0249* expression may be lethal for the bacterium.

*H. pylori* can incorporate cholesterol into its cell membrane (Wunder et al., 2006) and thus it appeared likely that the bacterium may have membrane raft-like structures in its outer membrane. Based on the known property of membrane raft components to partition to DRM fractions, our data indicate that *H. pylori* HP0248 is a membrane raft-associated protein (Figure 1, Supplementary Tables 3, 4). Bioinformatic analyses suggest that this protein does not have a signal sequence but does have the predicted transmembrane region (data not shown) required for the association of SPFH proteins with the bacterial membrane (Bramkamp and Lopez, 2015). Proteomic analyses of the DRM preparations (Table 1, Supplementary Tables 3, 4) also revealed a selective enrichment of OMPs, including several of which are important for adhesion e.g., BabA, HopB, HopC, HopQ. In *S. aureus*, DRMs were reported to contain proteins required for biofilm formation, signaling, attachment, and virulence (Lopez and Kolter, 2010). Similarly, selective packaging of outer surface lipoproteins into membrane rafts has been reported in *B. burgdorferi* (Toledo et al., 2014). We hypothesize that specific proteins, required for *H. pylori* interactions with target cells and/or colonization, may compartmentalize within the membrane raft domains of this pathogen.

Eukaryotic flotillin is found within membrane raft domains and is important for various cellular functions (Simons and Ikonen, 1997). We therefore speculated whether the flotillin-like protein, HP0248, renamed here as FLOT, may hold a similar importance for *H. pylori* physiology. Indeed, *H. pylori* ΔFLOT mutants displayed ~40% less cholesterol in their membranes (Figure 2A), thus indicating that the protein is involved in cholesterol accumulation by the bacterium. Although a cholesterol receptor has yet to be identified in *H. pylori*, previous experimental evidence suggested that cholesterol uptake in the bacterium is protein-mediated (Trampenau and Muller, 2003). It is therefore possible that *H. pylori* FLOT may be one protein required for the uptake of cholesterol within the *H. pylori* cell membrane. We hypothesize that through its stabilization of membrane raft domains, *H. pylori* FLOT may be important for cholesterol accumulation within its cell membrane. In turn, these domains may be required for the proper formation and functioning of the T4SS apparatus. Consistent with this hypothesis, we demonstrated that T4SS-dependent functions, as determined by cell scattering responses and CagA translocation, were significantly affected in *H. pylori* Δ*FLOT* mutants (Figures 3, 4, respectively). Furthermore, we showed that these mutants were also significantly affected in their ability to colonize mice in a bacterial strain-dependent manner (Figure 5). Similar strain-dependent effects on colonization have been reported for other *H. pylori* mutants (Grubman et al., 2010) and are most likely reflective of the enormous genetic diversity in this bacterial species (Falush et al., 2003).

In conclusion, the flotillin-like protein, HP0248 appears to be critical for *H. pylori* pathogenesis, as not only is this protein important for the induction of T4SS-mediated host cell responses, but also for colonization. Although we have shown that HP0248 is involved in the optimal delivery of CagA into host cells and complete induction of IL-8 immune responses, it would be of interest to directly show whether T4SS-dependent delivery of peptidoglycan to host cells is reduced in the absence of HP0248. Furthermore, it remains to be elucidated whether the reduced amounts of cholesterol in the *H. pylori* Δ*FLOT* bacteria, or a combination of reduced cholesterol and the absence of FLOT, account for the lower host cell responses and decreased colonization levels observed. The difficulty in separating the contribution of cholesterol and FLOT to the phenotypes observed during this study has raised further questions that will form the basis of future investigations. Are the observed HP0248-dependent phenotypes a result of lost interactions between FLOT and other *H. pylori* proteins that might complex within the membrane raft domain? Are these phenotypes an indirect effect of the reduction in membrane cholesterol, rather than through the direct loss of FLOT? Finally, is FLOT itself responsible for controlling integration of cholesterol into membrane microdomains? Whatever the relative contributions of cholesterol and FLOT, the fact that both have an association with DRMs identifies *H. pylori* membrane raft-like structures as critical to host-pathogen interactions during *H. pylori* infection. Finally, as cholesterol is a key metabolite in *H. pylori* physiology and plays a critical role in bacterial survival within the stomach, we propose that the biological pathways involved in accumulation of this sterol or its derivatives may be attractive targets for the design of new treatments against infection. Interestingly, *H. pylori*-infected subjects with high serum levels of total cholesterol and low-density lipoprotein cholesterol exhibited increased gastritis scores (Kucukazman et al., 2009). It is tempting to speculate whether increased levels of cholesterol within the gastric niche of these subjects might render *H. pylori* bacteria more pathogenic.

## Author contributions

MH and KD performed and analyzed experiments and drafted initial versions of the manuscript. AR designed and constructed suicide vectors for construction of mutants and analyzed the SPFH domain of HP0248. LW, SM, and BC assisted with acquisition and interpretation of data. LT assisted with the construction of vectors for expression of FLOT protein and generation of FLOT mutants. DS performed and analyzed all proteomic analyses. MK assisted with the design of experiments. RF conceived and coordinated the study and drafted the final version of the manuscript. All authors reviewed the results and approved the final version of the manuscript.

### Conflict of interest statement

The authors declare that the research was conducted in the absence of any commercial or financial relationships that could be construed as a potential conflict of interest.
