# Supplementary material for: A Helicobacter pylori Homolog of Eukaryotic Flotillin Is Involved in Cholesterol Accumulation, Epithelial Cell Responses and Host Colonization
Source: Front Cell Infect Microbiol. 2017 Jun 6;7:219. doi: 10.3389/fcimb.2017.00219 (PMC5460342; doi:10.3389/fcimb.2017.00219)
Supplement: Supplementary file 1 [file Table1.docx]

**Supplementary Table 1. Primers used in this study**

| **Primer name** | **Primer sequence** |
| --- | --- |
| **MH9** | GGGGACAAGTTTGTACAAAAAAGCAGGCTTAgggatccacttctttgtgcctatc |
| **MH10** | GGGGACCACTTTGTACAAGAAAGCTGGGTAtcattcttggcgcgcgatttggac |
| **MH1** | GGGGACAAGTTTGTACAAAAAAGCAGGCTTAATGCCCATTGATTTGAACGAACAT |
| **MH3** | GGGGACAACTTTGTATAGAAAAGTTGGGTGCCACAATGAGAATGTCTTGAATGA |
| **GmB4rF** | GGGGACAACTTTTCTATACAAAGTTGCTGGTACCCGGGTGACTAAC |
| **GmB3rR** | GGGGACAACTTTATTATACAAAGTTGTGGATCCCCGTGTCATTATTCC |
| **MH4** | GGGGACAACTTTGTATAATAAAGTTGCTAAAAGTCCAAATCGCGCGCCAAGA |
| **MH2** | GGGGACCACTTTGTACAAGAAAGCTGGGTACTATTAAGGCTCTTTAGTCTCGGCAAT |
| **M13** | TGTAAAACGACGGCCAGT (f)  TCACACAGGAAACAGCTATGAC (r) |
| **T7** | TAATACGACTCACTATAGGG (promoter)  GCTAGTTATTGCTCAGCGG (terminator) |
| **MH5** | CCTAATACGCCTAATAATGGG |
| **MH6** | GGCTCTTTAGTCTCGGCAAT |
| **GmFwd** | gacacgatgccaacacgacg |
| **GmRvs** | agggcctcgatcagtccaag |
| **LT8F** | GGGGACAAGTTTGTACAAAAAAGCAGGCTCAATGAAATTTTTGGATCAAG |
| **LT8R** | GGGGACAACTTTTGTATACAAAGTTGTATTTGAGCAAAAGAGGGGATC |
| **LT9F** | GGGGACAACTTTGTATACAAAAGTTGCATTAGTTAATGAACGCTTCTG |
| **LT9R** | GGGGACAACTTTGTATAGAAAAGTTGGGTGTTTTGGGGTGAGTTTCATCTC |
| **MH11** | GGGGACAACTTTTCTATACAAAGTTGCAATGCCCATTGATTTGAACGAAC |
| **MH12** | GGGGACAACTTTATTATACAAAGTTGTTTAAGGCTCTTTAGTCTCGGC |
| **LT10F** | GGGGACAACTTTGTATAATAAAGTTGCAGAGATTCAACCACAGCATGC |
| **LT10R** | GGGGACCACTTTGTACAAGAAAGCTGGGTATCACAACCAAGTAATCGCATC |
| **AG1F** | ACCACACGGCCACTACATGC |
| **MH13R** | ACCACACGGCCACTACATGC |
