# Supplementary material for: A Helicobacter pylori Homolog of Eukaryotic Flotillin Is Involved in Cholesterol Accumulation, Epithelial Cell Responses and Host Colonization
Source: Front Cell Infect Microbiol. 2017 Jun 6;7:219. doi: 10.3389/fcimb.2017.00219 (PMC5460342; doi:10.3389/fcimb.2017.00219)
Supplement: Supplementary file 2 [file Table2.docx]

| **HP**  **No.** | **Protein** | **Cytoplasmic** | **Cytoplasmic Membrane** | **Periplasmic** | **Outer Membrane** | **Extra-cellular** |
| --- | --- | --- | --- | --- | --- | --- |
| 0072 | Urease subunit B (UreB) | 100 *^a^* | 0 | 0 | 0 | 0 |
| 0073 | Urease subunit A (UreA) | 100 | 0 | 0 | 0 | 0 |
| 0248 | Flotillin-like protein | 89.6 | 5.1 | 2.6 | 0.1 | 2.6 |
| 1132 | ATP synthase F1, subunit beta | 91.2 | 8.8 | 0 | 0 | 0 |
| 1462 | Secreted protein involved in motility | 89.6 | 5.1 | 2.6 | 0.1 | 2.6 |
| 1563 | Alkyl hydroperoxide reductase (TsaA) | 100 | 0 | 0 | 0 | 0 |

**Supplementary Table 2.** Cellular locations of cytoplasmic proteins as predicted by the PSORTb program.

*^a^* Predicted locations are reported as percentages.
