# Supplementary material for: A Helicobacter pylori Homolog of Eukaryotic Flotillin Is Involved in Cholesterol Accumulation, Epithelial Cell Responses and Host Colonization
Source: Front Cell Infect Microbiol. 2017 Jun 6;7:219. doi: 10.3389/fcimb.2017.00219 (PMC5460342; doi:10.3389/fcimb.2017.00219)
Supplement: Supplementary file 4 [file Table4.docx]

**Supplementary Table 4. List of proteins migrating in the 40 kDa molecular weight range and common to DRM fractions from *H. pylori* WT**, ***∆FLOT* and *∆FLOT* (*FLOT+*) bacteria but absent from all DSM fractions. ^1^**

| HP number | Protein name | Accession number | Da | Mascot search score | Total peptides matched |
| --- | --- | --- | --- | --- | --- |
| HP0010 | 60 kDa Heat shock protein, chaperone (GroEL/HspB) | P42383 | 58285 | 108 | 10 |
| HP0019 | Chemotaxis protein (CheV1) | O24864 | 36735 | 30 | 2 |
| HP0045 | Nodulation protein (NolK) | O24886 | 35075 | 93 | 7 |
| HP0106 | Cystathionine γ-synthase (MetB) | P56069 | 41320 | 52 | 2 |
| HP0118 | Hypothetical protein | O24937 | 45336 | 20 | 1 |
| HP0176 | Fructose-biphosphate aldolase (Fba/Tsr) | P56109 | 33865 | 230 | 15 |
| HP0197 | S-adenosylmethionine synthase (MetK) | P56460 | 42678 | 40 | 2 |
| HP0254 | Outer membrane protein 8 (OMP8, HopG) | O25036 | 47526 | 38 | 5 |
| HP0267 | Adenosine deaminase | O25046 | 45798 | 68 | 3 |
| HP0393 | Chemotaxis protein (CheV3) | O25154 | 35665 | 51 | 1 |
| HP0554 | Hypothetical protein | O25280 | 37202 | 81 | 4 |
| HP0569 | GTP-binding protein (Gtp1) | O25293 | 40834 | 53 | 2 |
| HP0570 | Cytosol aminopeptidase (PepA) | O25294 | 54969 | 41 | 1 |
| HP0582 | Hypothetical periplasmic protein (TonB homolog) | O25304 | 37359 | 47 | 5 |
| HP0589 | 2-oxoglutarate-acceptor oxidoreductase subunit (OorA) | O25311 | 41596 | 26 | 2 |
| HP0631 | Hydrogenase (NiFe) small subunit (HydA) | O25348 | 42954 | 50 | 2 |
| HP0656 | Dehypoxanthine futalosine cyclase | O25370 | 43738 | 47 | 2 |
| HP0694 | Hypothetical protein | O25401 | 29996 | 150 | 5 |
| HP0825 | Thioredoxin reductase (TrxB) | P56431 | 34030 | 287 | 13 |
| HP0913 | OMP21 (HopB, AlpB) | O25571 | 57141 | 19 | 2 |
| HP1110 | Pyruvate ferredoxin oxidoreductase, α subunit (PorA) | O25738 | 45000 | 26 | 1 |
| HP1111 | Pyruvate ferredoxin oxidoreductase, β subunit (PorB) | O25739 | 35390 | 31 | 3 |
| HP1118 | γ-glutamyltranspeptidase (GGT) | O25743 | 61113 | 62 | 4 |
| HP1133 | ATP synthase γ chain (AtpG) | P56082 | 34221 | 121 | 4 |
| HP1177 | OMP27 (HopQ) | O25791 | 69991 | 55 | 4 |
| HP1335 | Predicted tRNA-specific 2-thiouridylase (MnmA) | O25893 | 38508 | 71 | 5 |
| HP1345 | Phosphoglycerate kinase (Pgk) | P56154 | 44915 | 60 | 4 |
| HP1349 | Hypothetical  periplasmic protein | O25904 | 44538 | 37 | 1 |
| HP1373 | Rod shape-determining actin-like protein (MreB) | O25925 | 37488 | 220 | 7 |
| HP1398 | Alanine dehydrogenase (Ald) | O25948 | 41256 | 196 | 8 |
| HP1554 | 30S ribosomal protein S2 (RpsB) | P56009 | 30839 | 61 | 2 |

^1^ Proteins were identified by LC-MS/MS using the QExactive mass spectrometer.
