# Supplementary material for: A Helicobacter pylori Homolog of Eukaryotic Flotillin Is Involved in Cholesterol Accumulation, Epithelial Cell Responses and Host Colonization
Source: Front Cell Infect Microbiol. 2017 Jun 6;7:219. doi: 10.3389/fcimb.2017.00219 (PMC5460342; doi:10.3389/fcimb.2017.00219)
Supplement: Supplementary file 5 [file DataSheet1.docx]

**SUPPLEMENTARY MATERIAL**

**A *Helicobacter pylori* Homolog of Eukaryotic Flotillin**

**Is Involved in Cholesterol Accumulation,**

**Epithelial Cell Responses and Host Colonization**

Melanie L. Hutton ^1¶#^, Kimberley D’Costa ^1#^, Amanda E. Rossiter ^1¶^, Lin Wang ^1^, Lorinda Turner ^1¶^, David L. Steer ^2^, Seth L. Masters ^3^, Ben A. Croker ^3¶^, Maria Kaparakis-Liaskos ^1^, Richard L. Ferrero ^1, 4*^

* Correspondence: Richard L. Ferrero, [Richard.Ferrero@Hudson.org.au](mailto:Richard.Ferrero@Hudson.org.au)

**Supplementary Methods**

**Expression and purification of recombinant HP0248.** *E. coli* bacteria were cultured in 5 mL of LB broth (100 μg/mL of ampicillin) and incubated at 37°C with shaking at 200 rpm to an A_600_ of 0.6. Bacteria from these starter cultures were then used to inoculate 250 mL LB broth cultures, which were grown to an A_600_ of 0.4. Expression was induced by the addition of 0.4 mM isopropyl β-D-1-thiogalactopyranoside (IPTG; Promega) for 16 h at room temperature. After expression, bacterial suspensions were pelleted and resuspended in 20 mL of Buffer A [50 mM Tris-HCL; pH 8.0, 100 mM NaCl, 0.5 % (v/v) Triton X-100 and protease inhibitors (Sigma)]. Bacteria were lysed by sonication and incubated with 0.1 mg/ml lysozyme for 20 min at room temperature prior to centrifugation at 6400 x g for 20 min at 4°C (Beckman J2-21M/E, USA). The insoluble pellets were resuspended in 7.5 mL of Buffer A, sonicated and centrifuged at 6400 X g for 20 min at 4°C. The pellets were resuspended in 15 mL of Buffer B (Buffer A without Triton-X or protease inhibitors) and sonicated on ice. MgSO_4_ (final concentration of 10 mM) was added to the pellets which were incubated for 20 min at 4°C prior to centrifugation at 12,000 X g for 20 min at 4°C. The resulting pellets were resuspended in 3.75 mL Buffer C (20 mM Tris-HCL; pH 8.0, 20 mM NaCl), sonicated and 3.75 ml of Buffer D [20 mM Tris-HCL; pH 8.0, 20 mM NaCl, 6M guanidine hydrochloride (Amresco, Ohio, USA)] was added. The final concentration of guanidine was adjusted to 6 M, prior to continuous mixing and incubation for 30 min at 4°C. Solubilized proteins were recovered following centrifugation at 17 000 x g for 30 min at 4°C. Supernatant fractions in 20 mM imidazole were loaded onto a Nickel column (GE Healthcare, Uppsala, Sweden) equilibrated with Buffer E (10 mM Tris-HCL, 150 mM NaCl), containing 20 mM imidazole (Sigma). Following an overnight incubation, the column was washed 3 times with Buffer E containing 20 mM imidazole. A step-wise elution was performed using Buffer E, containing increasing amounts of imidazole (100-500mM). The eluted protein was dialyzed overnight against PBS and its protein concentration measured by Qubit (Life Technologies). Polyclonal antibodies to HP0248 were generated by administering the purified recombinant protein to a New Zealand White rabbit (Walter and Eliza Hall Institute of Medical Research Antibody Facility; Bundoora, Melbourne, Australia). This anti-HP0248 serum (diluted 1:1000) was used in Western blot analyses.

**Proteomic analysis of *H. pylori* DRM and DSM fractions.** For MALDI-TOF, digested samples were co-spotted onto the MALDI target plate with matrix solution of 10 mg/ml a-cyano-4-hydroxycinnamic acid (Laser BioLabs, Sophia-Antipolis, France) in 50% acetonitrile, 0.1% trifluoroacetic acid. The samples were analyzed on an Applied Biosystems (Foster City, CA, USA) 4700 Proteomics Analyser MALDI TOF/TOF in reflectron mode with a mass range of 800 to 3500 Da, focus mass of 1400 Da at 1500 shots per spectra. The spectra were calibrated using the modified Porcine Trypsin Autolysis peaks: 842.4, 870.5, 1045.5, 1970.9, 2211.1, 2235.1, 2239.1, 2283.1, 2289.1, as internal calibrants matching a minimum of 4 peaks at 0.4 Da tolerance and equal weighted fit. Failing these criteria, the spectra were calibrated by the default calibration which was updated by the plate model method immediately prior to sample acquisition. The spectra were processed with peak detection of 20000 resolution at 1500 Da, minimum signal to noise threshold of 5 and monoisotopic peak de-isotoping. The 4700 Series Explorer software selects the 12 most intense peptides. These were selected as precursor masses for MS/MS analysis and were acquired in an order of decreasing intensity, with an exclusion list of porcine modified trypsin autolysis peaks, sodium and potassium adducts. MS/MS analysis was carried out in reflector mode with spectra summed to 2500 shots/spectrum and spectra were processed with baseline subtraction of peak width 10, peak detection of 5000 resolution at 1000 Da and monoisotopic peak detection. The PMF and MS/MS data were compiled by the GPS explorer software Vers. 3 (build 311) (Applied Biosystems, Foster City, CA, USA) and searched against an in-house database compiled from *H. pylori* genomes downloaded from the ExPASy FTP site (ftp.expasy.org) using the MASCOT search engine (version 1.9, Matrix Science Inc., London, UK) with all taxonomy selected. The following search parameters used were: missed cleavages, 1; peptide mass tolerance, ± 50 ppm; peptide fragment tolerance, ± 0.1 Da; peptide charge, 1+; fixed modifications, carbamidomethyl; variable modification, oxidation (Met) and the top 5 matches reported. Scores were considered significant when above the MASCOT-generated probability-based Mowse score minimum threshold. Matched proteins below this threshold were manually verified by MSMS matching spectra if available, or otherwise were excluded. Further manual verification was performed by comparison of the error distribution of matched peptides.

LC-MS/MS was performed using a HCT ULTRA ion trap mass spectrometer (Bruker Daltonics, Bremen, Germany), coupled online with an RSLC nano HPLC (Ultimate 3000, Dionex Corporation, SunnyBrook, CA, USA). Samples were injected onto a Dionex pepmap100, 75 µm id, 100Ǻ pore size, reversed phase nano column with 95% buffer A (0.1% (v/v) formic acid) at a flow rate of 300 nl/minute. The peptides were eluted over a 30-minute gradient to 70% buffer B (80% (v/v) acetonitrile, 0.1% (v/v) formic acid). The eluent was nebulized and ionized using the Bruker ESI electrospray source via the nanoflow ESI sprayer with a capillary voltage of 4000V, dry gas at 200ºC and flow rate of 5.0l/min and nebulizer gas at 6 psi. Peptides were selected for MS/MS analysis in autoMSn mode with smart parameter settings selected with a target mass of 900m/z and active exclusion released after 1 min. Data from LC-MS/MS analysis were exported in the Mascot generic file format (*.mgf) and searched against an in-house database, as above. The following search parameters were used: enzyme specificity, trypsin; missed cleavages, 1; peptide mass tolerance, ± 0.6 Da; peptide fragment tolerance, ± 0.3 Da; peptide charge, 2+ and 3+; fixed modifications, carbamidomethyl; variable modification, oxidation (Met).

To identify proteins in DRM and DSM fractions from *H. pylori* WT, *∆FLOT* and *∆FLOT* (*FLOT+*) bacteria, sections of SDS-PAGE gel corresponding to a molecular weight of approximately 40 kDa were excised. These sections of gel (approximately 1 cm high and spanning the width of the gel) were sliced into 6 pieces and each trypsin digested prior to analysis. LC-MS/MS was performed using the QExactive mass spectrometer (Thermo Scientific, Bremen, Germany) coupled online with an RSLC nano HPLC (Ultimate 3000, Thermo Scientific, Bremen, Germany). Samples were concentrated on a 100 µm, 2 cm nanoviper pepmap100 trap column with 95% buffer A (0.1% Formic acid) at a flow rate of 15 µl/min. The peptides were then eluted and separated with a 50 cm Thermo RSLC pepmap100, 75 µm id, 100Ǻ pore size, reversed phase nano column with the following gradient: 90 % buffer A (0.1 % Formic acid; 30 min), 30 % buffer B (80 % Acetonitrile, 0.1 % formic acid; 25 min) and 40 % buffer B (30 min). The flow rate was 300 nl/min. The eluant was nebulized and ionized using the Thermo nano electrospray source with a distal coated fused silica emitter (New Objective, Woburn, MA, USA) with a capillary voltage of 1900 V. Peptides were selected for MS/MS analysis in Full MS/dd-MS^2^ (TopN) mode with the following parameter settings: TopN 10, resolution 17500, MSMS AGC target 1e5, 60 ms Max IT, NCE 27 and 3 m/z isolation window. Underfill ratio was at 10 % and dynamic exclusion was set to 15 seconds. For each sample, pooled data from 6 gel slices were exported in the Mascot generic file format (*.mgf) and analyzed as above.
