# Supplementary figures and images for: A Helicobacter pylori Homolog of Eukaryotic Flotillin Is Involved in Cholesterol Accumulation, Epithelial Cell Responses and Host Colonization
Source: Front Cell Infect Microbiol. 2017 Jun 6;7:219. doi: 10.3389/fcimb.2017.00219 (PMC5460342; doi:10.3389/fcimb.2017.00219)

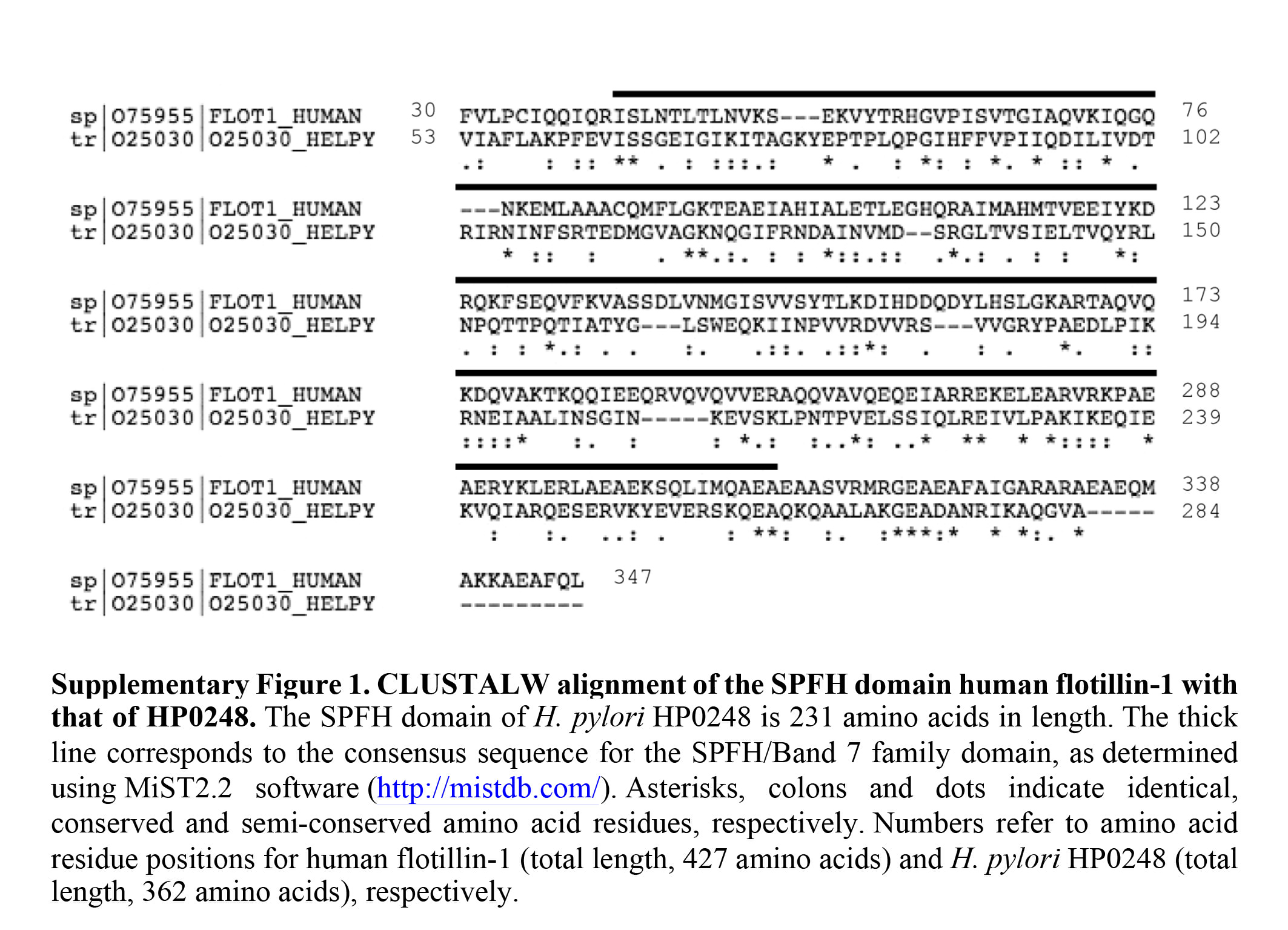

Supplement: Supplementary file 6 [file Image1.tif]

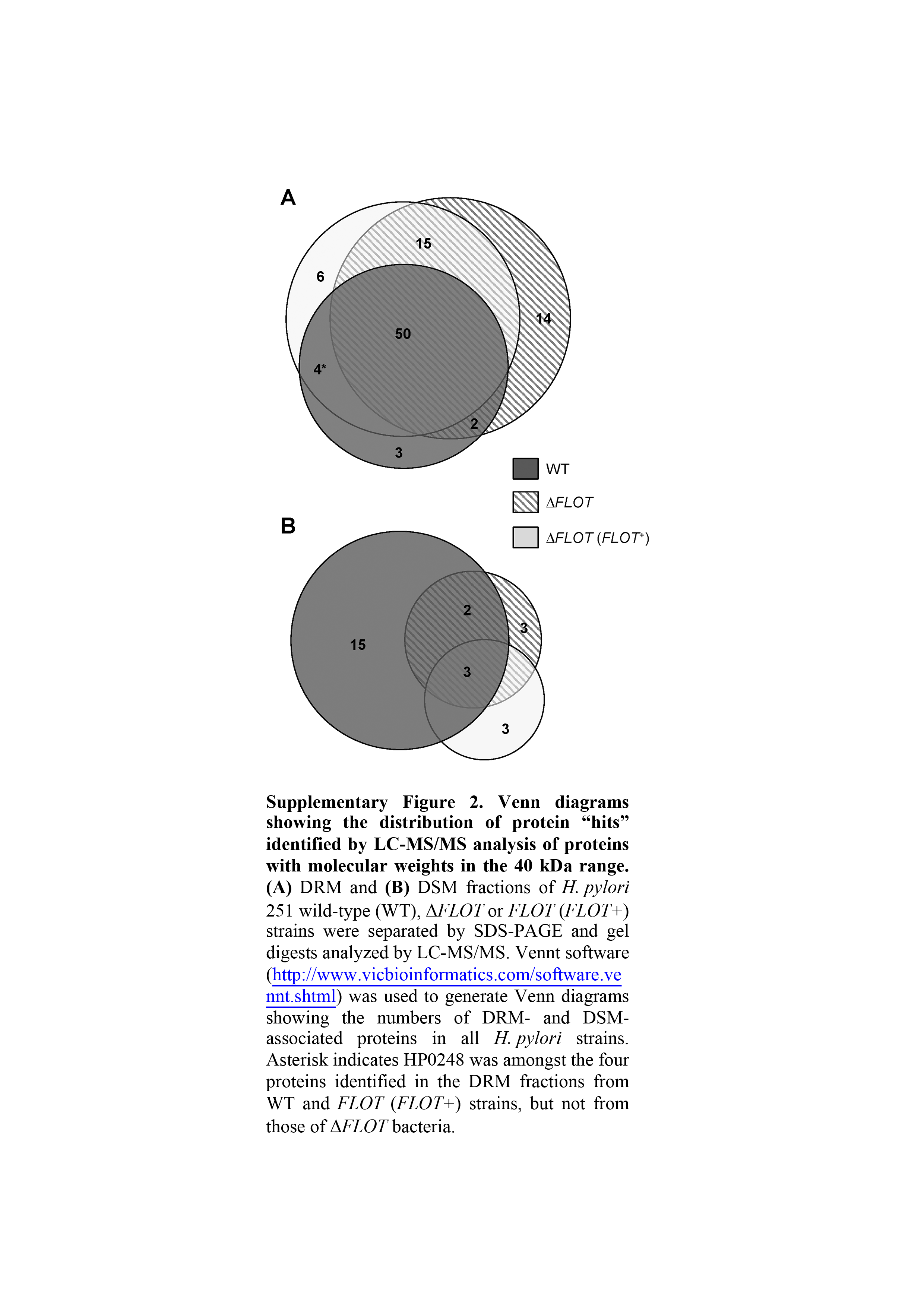

Supplement: Supplementary file 7 [file Image2.tif]
